# Cellular mechanisms of reverse epithelial curvature in tissue morphogenesis

**DOI:** 10.3389/fcell.2022.1066399

**Published:** 2022-11-28

**Authors:** Yiran Wang, Daniel Stonehouse-Smith, Martyn T. Cobourne, Jeremy B. A. Green, Maisa Seppala

**Affiliations:** ^1^ Centre for Craniofacial and Regenerative Biology, Faculty of Dentistry, Oral and Craniofacial Sciences, King’s College London, London, United Kingdom; ^2^ Department of Orthodontics, Faculty of Dentistry, Oral and Craniofacial Sciences, King’s College London, London, United Kingdom

**Keywords:** reverse curves, epithelial bending, apical/basal constriction, actomyosin, extrinsic factor

## Abstract

Epithelial bending plays an essential role during the multiple stages of organogenesis and can be classified into two types: invagination and evagination. The early stages of invaginating and evaginating organs are often depicted as simple concave and convex curves respectively, but in fact majority of the epithelial organs develop through a more complex pattern of curvature: concave flanked by convex and *vice versa* respectively. At the cellular level, this is far from a geometrical truism: locally cells must passively adapt to, or actively create such an epithelial structure that is typically composed of opposite and connected folds that form at least one s-shaped curve that we here, based on its appearance, term as “reverse curves.” In recent years, invagination and evagination have been studied in increasing cellular detail. A diversity of mechanisms, including apical/basal constriction, vertical telescoping and extrinsic factors, all orchestrate epithelial bending to give different organs their final shape. However, how cells behave collectively to generate reverse curves remains less well-known. Here we review experimental models that characteristically form reverse curves during organogenesis. These include the circumvallate papillae in the tongue, crypt–villus structure in the intestine, and early tooth germ and describe how, in each case, reverse curves form to connect an invaginated or evaginated placode or opposite epithelial folds. Furthermore, by referring to the multicellular system that occur in the invagination and evagination, we attempt to provide a summary of mechanisms thought to be involved in reverse curvature consisting of apical/basal constriction, and extrinsic factors. Finally, we describe the emerging techniques in the current investigations, such as organoid culture, computational modelling and live imaging technologies that have been utilized to improve our understanding of the cellular mechanisms in early tissue morphogenesis.

## Introduction

Epithelial morphogenesis relies on complex and dynamic cellular mechanisms and plays an important role during development of multiple vertebrate structures (Pispa and Thesleff, 2003; Davidson, 2012; Fouchard et al., 2020). In adult tissues, epithelial morphogenesis continues to play a part in maintaining a homeostatic environment and potential for regeneration that relies on function of stem cells and tissue remodeling (Gehart and Clevers, 2019; Nikolaev et al., 2020). In cancer progression, when the self-renewal of cells in a tissue undergoes a loss of control or the apicobasal mechanical tension becomes imbalanced, normal epithelial morphogenesis can be disrupted and become susceptible to transformation into full-blown carcinomas (epithelium-derived tumour) (Radtke and Clevers, 2005; Messal et al., 2019). Therefore, understanding the cellular mechanisms of epithelial bending during embryonic development can also shed further light on understanding the basic principles of the role of epithelial morphogenesis in pathological disease processes as well as in organ and tissue regeneration.

In organ development, epithelial morphogenesis fundamentally originates from bending of the epithelium, which is then followed by more complex stages of organogenesis. Although the gross histological appearance of these stages has been well described, there is still much to learn about how tissue morphogenesis is orchestrated at the cellular level. These mechanisms include cell shape change, positional changes of cells in relation to one another and how epithelial cells communicate with the surrounding tissues as they integrate to form a functional unit (Pearl et al., 2017).

**FIGURE 1:**
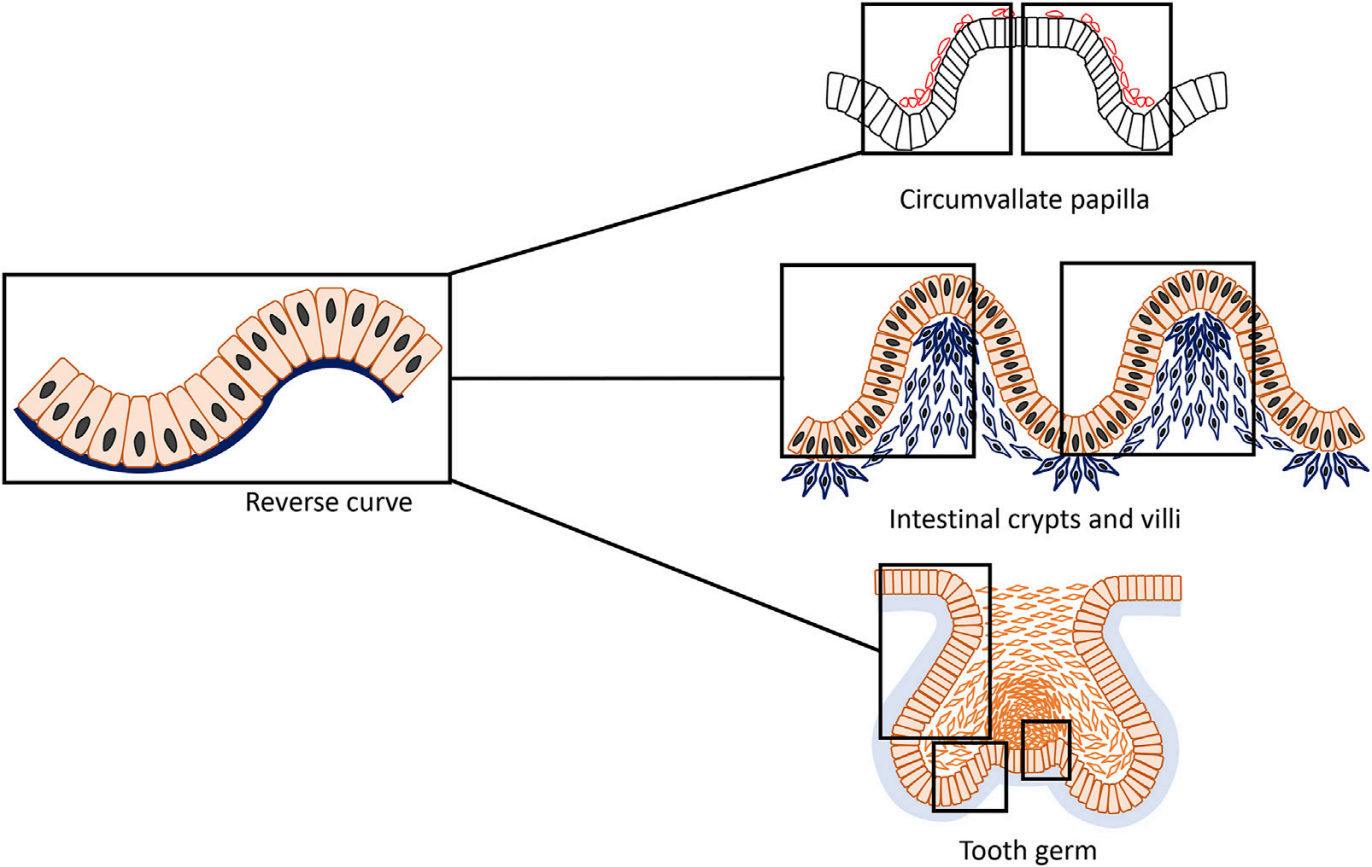
An epithelial structure that is typically composed of connected concave and convex with opposite directions form an s-shaped curve that can be termed “reverse curve.” It can be considered as one of the elementary units of epithelial morphogenesis and can appear for instance as isolated or repetitive phenomenon in different organs such as the developing mouse circumvallate papilla, small intestine and tooth. During formation of the circumvallate papilla, characteristically two adjacent reverse curves form a mirror image pattern; in the small intestine, reverse curves appear in a repetitive pattern giving rise to the crypts and villi; whereas in tooth development, reverse curves are found in a more complex and diverse mode.

Essentially, two classical and simplest types of epithelial bending have been described which leads to opposite directions of growth and folding: invagination and evagination (Davidson, 2012; Pearl et al., 2017). Invagination is characterized by an epithelial layer folding inwards towards the underlying tissue, such that the apical (superficial) surface of the cells usually forms the inner, concave surface, whilst the basal surface of these cells forms the outer ring of the early epithelial organ (Lewis, 1947). In contrast, evagination occurs outwards from the epithelial surface and during folding, the apical side of cells forms the exterior side of the protrusion and the basal area of the cells lie internally giving rise to the smaller side of the curvature (Sinn and Wittbrodt, 2013). Invagination and evagination are often thought of as representing simple concave or convex curves with most prior research considering them as entirely separate processes and concentrating on the molecular mechanisms that guide them. However, epithelial organogenesis in fact consists of more convoluted patterns of curvature (Shyer et al., 2013; Li J. et al., 2016; Adhikari et al., 2017; Sumigray et al., 2018; Yamada et al., 2019; Li et al., 2020; Zhang et al., 2020; Kim et al., 2021): reverse curves characterized by concave-shape flanked by convex or vice versa (Figure 1). Although our understanding of cell behavior has improved, much remains to be elucidated about how cells behave collectively to generate sophisticated tissue curvatures and how gene regulatory networks integrate with their physical behaviors.

This review aims to provide examples of mainstream cellular systems, how they are guided by various intrinsic and extrinsic factors during epithelial bending as well as introduce experimental models that characteristically exhibit reverse curves during morphogenesis and organogenesis. Furthermore, we attempt to provide a summary of potential mechanisms that might be involved in formation of reverse curvature and present an outlook of emerging techniques that may improve our understanding of the cellular mechanisms underlying early tissue morphogenesis.

## Cellular motifs in monolayer epithelial bending

Two important cellular events, cell shape change and cell rearrangement, have been shown to play key roles in epithelial morphogenesis that ultimately drive the well-described changes in tissue architecture and define the various stages of organogenesis. The importance of actomyosin in mediating cell shape changes and cell rearrangements in both apical and basal constriction has been well-characterized in a number of tissue models (Clark et al., 2007; Martin et al., 2009; Mason et al., 2013; Gutzman et al., 2015). Elementary cell behaviours contributing to morphogenesis have been described, namely cellular motifs including vertical telescoping movement, canopy contraction, apical and basal constriction and buckling by constrained growth (Pearl et al., 2017; Lemke and Nelson, 2021). These processes work actively or passively and in collaboration to subsequently drive either epithelial invagination or evagination (Gutzman et al., 2008; Martin et al., 2009; Mammoto et al., 2011; Mason et al., 2013; Yamada et al., 2019). At the earliest stages of vertebrate epithelial organ morphogenesis, they commonly form a single sheet of cells that then stratifies to consist of multiple cell layers or become pseudostratified with different types of cell shapes that falsely provide the appearance of multiple layers. Despite the fact that most of the described mechanistic theories are more relevant to stages of monolayer epithelial morphogenesis and are limited to only a few models and types, it is still beneficial to collate these investigations and highlight the existence and importance of reverse curves in more complex tissue systems and later phases of organ morphogenesis.

### Actomyosin-involved apical constriction during epithelial invagination

**FIGURE 2:**
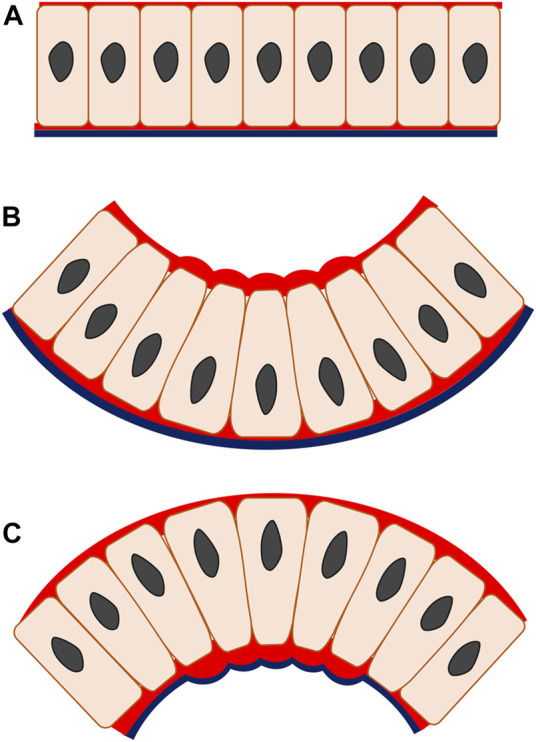
Apical and basal constriction seen in monolayer epithelium. In apical constriction **(B)**, the apical width of the cell is reduced, driven by the actomyosin enrichment on the apical side, enabling cell shape change from columnar to wedged. The epithelial construct changes from a flat layer **(A)** to fold inwards **(B)**, producing invagination. In basal constriction **(C)**, the reverse applies. The basal width of the cell is reduced due to actomyosin accumulation on the basal side, generating wedged-shape cells and outward bending epithelium termed as evagination. Red, actomyosin; black line, basal lamina.

Most epithelial bending mechanisms initiate as a localized cell shape transition from columnar to wedge-shaped and, depending on which side of the cell this takes place, results from either apical or basal constriction (Pearl et al., 2017). These cell shape transitions are driven by actomyosin, a major intrinsic effector that represents a fundamental component of the cytoskeletal system and functions to effect epithelial cell shape change from spindle to wedge (Martin et al., 2009; Mason et al., 2013). The formation of wedge-shaped cells relies on an actomyosin-dependent mechanism of apical constriction that appears in the epithelial invagination and is characterized by a reduction in the apical width of the cell whilst cell volume, or at least its basal width, remains constant, resulting from increased tension on the apical side (He et al., 2014) (Figures 2A,B). Characteristic enrichment of cytoskeleton component F-actin and motor protein myosin II is also found apically and supplemented with the contributing functions of cytoplasm and intercellular adhesive junctions, which give rise to the wedged cell shape that triggers the invagination (Martin et al., 2009; Mason et al., 2013). The specific principle of invagination is that when the actin-binding protein myosin II is active, the actin filaments become translocated along each other. This in turn, produces cytoskeletal tension and consequently regulates cell shape, causing the entire tissue to fold inwards (Clark et al., 2007). To maintain cell volume, cell height increases and/or basal expansion occurs. Often, this involves basal relaxation whereby the basal actomyosin network is actively disassembled, as observed during epithelial invagination of the chick otic placode (Jidigam et al., 2015). Some degree of basal relaxation is certainly required, as shown by the blocking of invagination by basal activation of myosin II during *Drosophila* gastrulation (Krueger et al., 2018). Actomyosin-driven apical constriction is indeed sufficient to produce embryo-scale inward buckling in the *Drosophila* model indicating a relatively passive (relaxed) role for the basal parts of the cells (Fierling et al., 2022). Another invagination mechanism, basal wedging, in which nuclei move basally to expand the bases of the narrow cells of the early neuroepithelium in a coordinated version of interkinetic nuclear migration, may or may not involve the actomyosin (microfilament) cytoskeleton (Schoenwolf et al., 1988; Ybot-Gonzalez and Copp, 1999).

### Actomyosin-associated basal constriction in epithelial evagination

In basal constriction, the cell width is reduced on the basal side of the cell and the subsequent cell shape transformation from columnar to wedge-shaped is followed by an outward folding of epithelial tissue, creating an evaginated geometry. This process has not been described as fully as apical constriction (Figures 2A,C). As the basal width of the cell contracts, tension rises on the basal surface during evagination of the mouse optic vesicle (Svoboda and O'Shea, 1987). At this stage, F-actin is localized intensely at the basal surface and knock-out of myosin II impairs the F-actin basal distribution and evagination morphogenesis in the zebrafish neural system (Gutzman et al., 2008; Gutzman et al., 2015).

Extracellular matrix proteins such as laminin and focal adhesion kinase (FAK), key regulators of cell shape change, cell migration, polarity and adhesion are linked to actomyosin-dependent basal constriction (Parsons et al., 1994; Miner and Yurchenco, 2004; Vicente-Manzanares et al., 2009; Schaller, 2010). Laminin, a secreted protein, is an essential part of the basal lamina mediating the cytoskeleton of overlying cells through integrin. An absence of laminin leads to a failure of basal constriction and epithelial evagination (Gutzman et al., 2008). The other influencer FAK, is a non-receptor tyrosine kinase interacting with multiple integrins and activating myosin II by phosphorylation to regulate cell shape, adhesion and migration and is therefore more crucial for basal constriction during epithelial evagination (Gutzman et al., 2018; Yamada et al., 2019; Zhang et al., 2020).

### Vertical telescoping and multicellular systems without basal wedging during epithelial invagination

Although the majority of epithelial invagination begins with apical constriction, there are other mechanisms that have differing coordinated multicellular patterns, such as the vertical telescoping cell movement seen in epithelial invagination of mammalian salivary glands and teeth (Li et al., 2020). In the salivary gland especially, cells towards the periphery of the placode migrate vertically with apical and centripetal protrusion, climbing up over the central neighbour cells to push them downwards, and forming inward folding of the epithelium to initiate an invagination.

**FIGURE 3:**
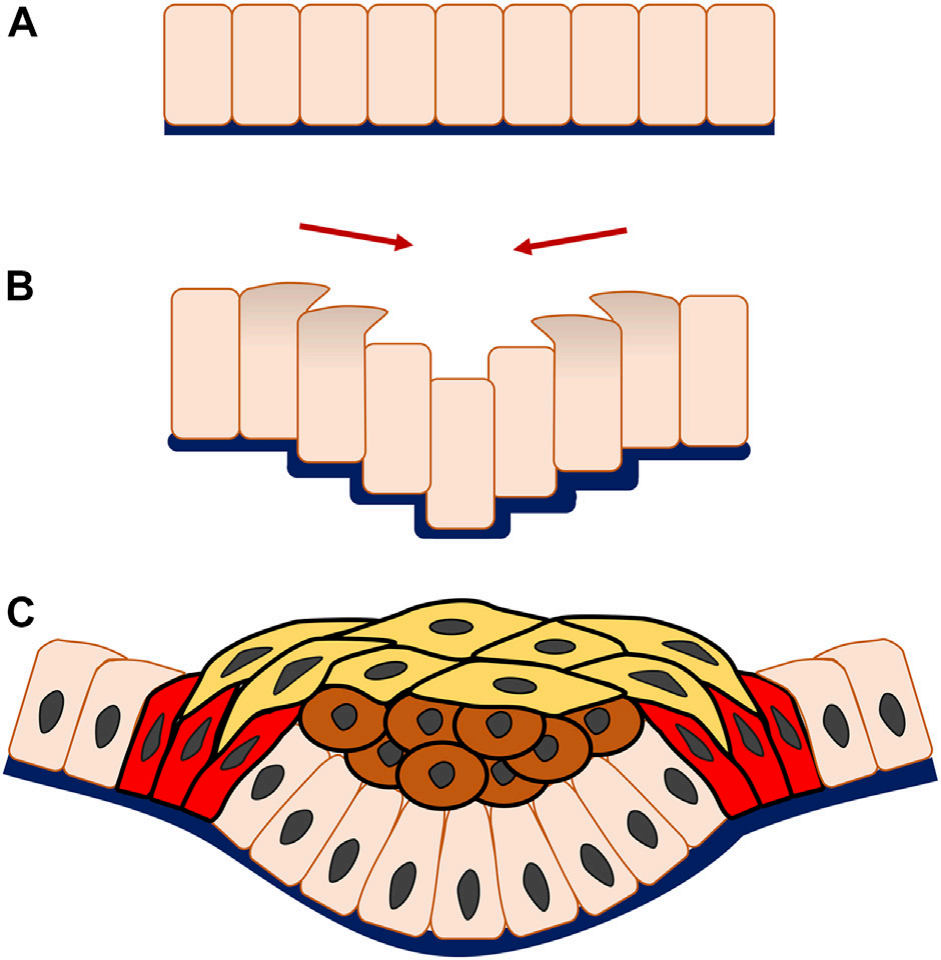
Multicellular system of epithelial invagination in early tooth development. As the invagination begins, cells at the edge of the monolayer **(A)** migrate vertically with apical and centripetal protrusion **(B)**, making the central adjacent cells telescope downwards, thereafter known as vertical telescoping movement. Interestingly, this occurs without cell shape change and triggers the epithelial layer to bend inwards and form an invagination. Subsequently, the multicellular invagination is formed by stratification of the central suprabasal cells (brown cells), horizontal shrinking driven by cell intercalation of the superficial suprabasal cells (yellow cells) and canopy contraction of the basal epithelial cells (red cells) **(C)**.

Progression of epithelial morphogenesis from a monolayer to multiple layers is associated with an increase in mechanistic complexity through the addition of cell layers and interaction. For instance, during invagination a replenished and stratified placode containing a great number of both suprabasal and basal cells may arise or additional structures form, such as a hollow pit with a basal layer and few suprabasal cells, as is seen in formation of the salivary gland and hair follicle. During the process of epithelial invagination in early tooth development, vertical telescoping takes place similar to the cell movement observed in salivary gland organogenesis. What distinguishes tooth organogenesis is that frank stratification occurs but is physically constrained by canopy contraction, that is the horizontal constriction of superficial a suprabasal cell layer through cell intercalation (Panousopoulou and Green, 2016) (Figure 3).

### Extrinsic factors during epithelial bending

In addition to previously described mechanisms responsible for epithelial invagination and evagination, epithelial movement and morphogenesis can also be influenced by the surrounding cellular context or external environment. For instance, during tooth organogenesis, following initial invagination to the so-called bud stage, the underlying neural crest-derived mesenchyme encompasses the epithelial placode tightly and communicates reciprocally with the invaginating epithelium (Pispa and Thesleff, 2003). It has been suggested that at the early stage of mouse tooth development, when epithelial invagination occurs, the mesenchyme functions to constrain the epithelium as proliferation increases its planar area, forcing it to fold by buckling and this idea has been supported by a number of computational modelling papers (Mammoto et al., 2011; Takigawa-Imamura et al., 2015; Marin-Riera et al., 2016, 2018; Morita et al., 2016). Experimental inhibition of proliferation has shown that most invagination to at least cap stage is epithelially-driven (Yamada et al., 2019; Li et al., 2020), but splaying of the structure laterally upon mesenchyme removal from explants also demonstrates that the overall width of the cap and bell stage structures are indeed constrained by the surrounding mesenchyme (Morita et al., 2016; Marin-Riera et al., 2018). Modelling has also been used to illustrate villi morphogenesis in the chick and murine gut. This process involves bulging of the endodermal epithelium, which appears to depend on compression exerted by the smooth muscle layer that in turn, differentiates from the underlying mesenchyme (Shyer et al., 2013; Huycke and Tabin, 2018). Experimental evidence shows, however, that while this mechanism does occur in the chick gut, it is not so clear in the mouse. However, the mechanism in mouse also appears to be non-autonomous to the epithelium: villi arise due to focal condensations of underlying mesenchymal cells (Hughes et al., 2018).

The biomechanical properties of epithelium itself are indispensable in responding to extrinsic stimulation in the formation of epithelial bending. Myosin II depletion decreases the stiffness of neuroepithelium and causes the epithelium to become more deformable under external force during neural tube closure in Xenopus (Rolo et al., 2009). It has also been suggested that neuroepithelial bending observed during brain ventricle lumen expansion and hindbrain morphogenesis in the zebrafish is dependent upon epithelial relaxation, a term used to describe the contractile state of the neuroepithelium (Gutzman and Sive, 2010). Consistent with the results of neural tube closure in Xenopus, the overaction of myosin II in zebrafish neural system development impaired neuroepithelium extensibility, causing the failure of neuroepithelium bending and producing a dysmorphic brain ventricle amongst other defects.

## Reverse curves in experimental animal models

While we have so far focused on separately discussing the cellular mechanisms of invagination and evagination initiating from a single epithelial layer, these two morphological structures can also together contribute to more convoluted stages of epithelial organ development when two opposite epithelial bendings occur abreast actively or passively that, as previously mentioned, we hereby describe as “reverse curves. Little attention has been paid to them in the literature, however their appearance is essential for progression of the most epithelial organs to more advanced and elaborate stages of morphogenesis. Here we review some experimental models that characteristically contain this phenomenon of reverse curvature during organogenesis, such as circumvallate papilla of the tongue, crypts and villi of the intestine, and the developing tooth germ. Although these models demonstrate individual traits in their development, they also share many commonalities. Using each of the models as an example, we demonstrate how reverse curves develop and how they connect invaginated and evaginated or oppositely bent epithelium.

### Circumvallate papillae morphogenesis in the tongue

Circumvallate papillae (CVP), one of the three gustatory papillae, are located at the dorsal posterior surface of the mouse tongue. At the gross level, these are the largest papillae and contain a high density of taste buds, which are an essential constituent to the structure and function of the tongue (Jitpukdeebodintra et al., 2002; Lee et al., 2006).

**FIGURE 4:**
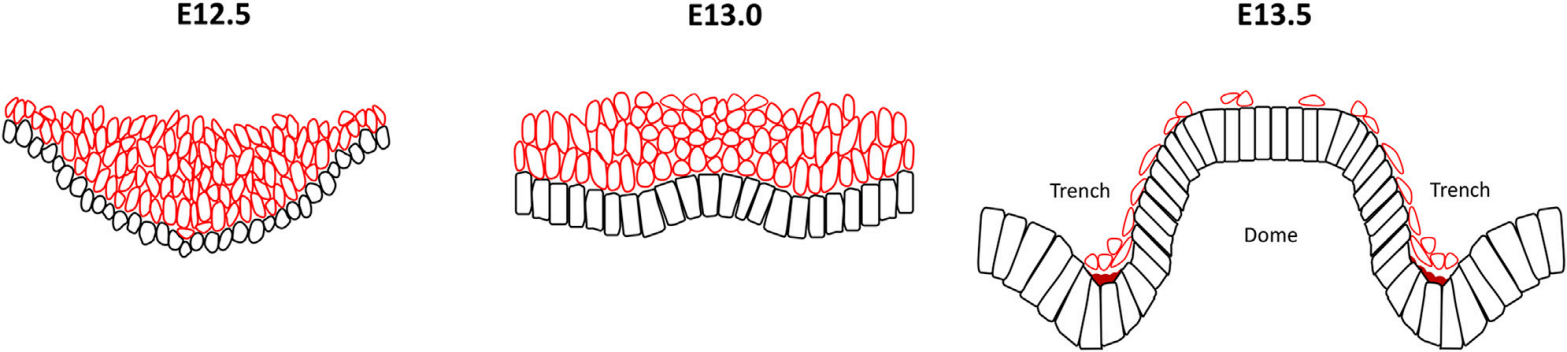
Reverse curve morphogenesis in the circumvallate papillae of the tongue. At E12.5 the developing circumvallate papillae consists of an invaginated placode with round epithelial basal cells, elongated suprabasal cells and actomyosin enrichment at the basal surface of the basal cells. At E13.0, inwards folding changes to outwards bend in the centre of the placode with a low cell density mesenchyme localized under the evagination. The wedged-shape basal cells in the medial region exhibit basal constriction with actomyosin accumulation whilst the overlying suprabasal cells become more ovoid shape and cells in the lateral part maintain a columnar morphology. At E13.5, the stratified epithelium gives rise to two bilateral trenches with a central dome that exhibit basal constriction of the basal epithelial cells present at the lateral side of the trench and medial region of the dome. In contrast, the basal epithelial cells in the median trench region show apical constriction. Additionally, a notable reduction in the number of suprabasal cells is seen. Black cells, basal cells; red cells, suprabasal cells; dark red region, actomyosin accumulation.

CVP have a distinctive developmental process initiated from a multilayer invaginated placode, first seen at E12.5 (Jitpukdeebodintra et al., 2002; Zhang et al., 2020). This is followed by a reverse in folding outwards at the centre of the placode with a low cell density of mesenchyme confined under the evagination. By E13.5, this evagination gradually becomes a domed monolayer or pseudostratified layer bound by invaginated trenches where two reverse curves then become evident as a mirror image pattern (Lee et al., 2006) (Figure 4). Compared with the crypt–villus structure in the intestine or base of the developing tooth germ, it is unusual that growth orientation is overturned from the original median concave to a convex bend and consequently the suprabasal cells exist first then disappear. The cellular mechanisms of the reverse curves in this example are likely to be attributed intrinsically by the actomyosin system, cell shape changes clearly reflecting how basal constriction forms in the evaginated central dome and the apical constriction in the invaginated bilateral trenches (Zhang et al., 2020).

At E12.5, CVP epithelial cells exhibit a rounded morphology and the majority of suprabasal cells are elongated. Later at E13.0, the basal cells of the medial region are wedge-shaped with reduced basal widths. Cell shortening has not been observed, meanwhile, the suprabasal cells overlying those basal constricted cells become ovoid and cells in the lateral regions maintain a columnar morphology. Moreover, the angulation of suprabasal cells showed that most of them are not parallel to the plane of the flanking epithelium which would exclude the canopy intercalation theory discussed earlier. By E13.5 the basal epithelial cells lateral to the trench and the medial region of the dome become spindle shaped due to an increased apical/basal width ratio, interpreted as basal constriction. In contrast, basal epithelial cells in the median trench region were characterized by apical constriction with a decreased apical/basal width ratio. The total number of suprabasal cells also decreased dramatically during this stage (Jitpukdeebodintra et al., 2002; Zhang et al., 2020).

The formation of the reverse curves in CVP are mostly activated by the actomyosin system (Zhang et al., 2020). Early in development at E12.5, F-actin locates basally within the basal cells. This accumulates at the basal and medial region of the placode by E13.0 followed by widespread enrichment both in the apical side of the trench and the basal side of the dome, corresponding to the basal constriction in the median trench and apical constriction in the median dome respectively. In addition, phospho-Myosin light chain II (pMLC) overlaps with F-actin which accumulated at the basal surface of basal cells at E12.5 and E13.0, indicating active actomyosin involvement during early CVP morphogenesis. Mouse tongue explant culture has shown that F-actin enriches at the basal side of dome cells and the apical side of trench cells in concert with the F-actin distribution of *in vivo* results. The inhibition of myosin II in mouse tongue explant culture also produces morphological defects with reduced basal constriction in the dome and impaired apical constriction in the trench region. Inhibition of FAK damages the dome more widely due to reduced basal constriction with F-actin severely reduced at corresponding sites (Zhang et al., 2020). The suprabasal intercalation cellular motifs which account for the morphogenesis of other models are invalid in the reverse curves seen during CVP morphogenesis when considering the dramatic decline in the number of suprabasal cells and differential E-cadherin expression (Jitpukdeebodintra et al., 2002; Zhang et al., 2020). External forces from differential cell proliferation of the epithelium could also be excluded as no significant difference in proliferation or apoptosis has been observed in CVP epithelium and the role of mesenchyme appears ambiguous (Jitpukdeebodintra et al., 2002; Zhang et al., 2020). Beyond cellular mechanisms, molecular mechanisms are also a critical regulator in reverse curves. Shh signaling activity is continuously found from the early placode to the dome-shape at E13.5 (Hall et al., 1999; Liu et al., 2004; Lee et al., 2006; Iwatsuki et al., 2007). Expression and culture experiments support that Shh signaling regulates dome and trench formation by actomyosin-dependent epithelial folding through invagination and evagination (Zhang et al., 2020).

### Crypt–villus structure in the intestine

Human and mouse intestinal morphogenesis establishes a unique microarchitecture characterized by spatially and repetitively organized crypts and villi, which are indispensable for the preservation of intestinal homeostasis and efficient nutrient absorption. Crypt–villus structure in the intestine is a clear example of iterative reverse curves with adjoining evagination and invagination, respectively. The cellular mechanisms can be separated into an extrinsically influenced evagination, whilst invagination is regulated internally through the actomyosin system (Tan and Barker, 2014; Sumigray et al., 2018).

**FIGURE 5:**
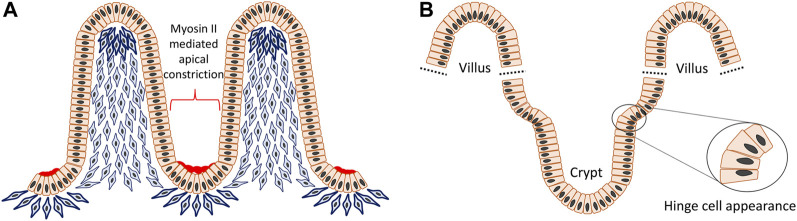
Reverse curve morphogenesis of crypt-villus structure in the intestine. The villi are evaginations toward the intestinal lumen and the crypts are invaginations between villi. In the beginning, mesenchymal cells underneath the flat pseudostratified epithelium condense at intervals and then aggregate to move towards the lumen and support the superficial epithelium to protrude to form the tip of the villi. The villus height is increased by cell proliferation, however at the same time some epithelial cells undergoing mitosis, experience apical constriction with the accumulation of myosin II and actin and contribute to formation of the crypts **(A)**. Also, at the crypt-villus junction there is an interesting region formed by so called “hinge cells” that adopt a wedge shape through basal constriction **(B)**.

The villi or microvilli develop into finger-shaped protrusions towards the intestinal lumen, increasing the overall surface area of the small intestine. This apparent epithelial evagination is a collaborative outcome of extrinsic mesenchymal extrusion and multiple signaling molecules. At the beginning, mesenchymal cells underneath the flat pseudostratified intestinal epithelium condense at intervals and then these clustered mesenchymal cells migrate toward the lumen and force the superficial epithelium to protrude and form the tips of the villi (Figure 5A) (Spence et al., 2011a; Walton et al., 2016). Although crypts can also develop independently in gut organoids, during intestinal crypt-villus morphogenesis they adopt a flask shape at intervals between villi in the epithelial layer overlying the mesenchyme (Sato and Clevers, 2013; Sumigray et al., 2018). The crypt occurs through internalized mitotic cell rounding followed by apical constriction with the accumulation of myosin II and actin, as well as compression from the bilateral clustering of mesenchymal cells, as demonstrated both *in vivo* and mathematic modelling (Freddo et al., 2016; Sumigray et al., 2018). After primary intestinal morphology is established, the villi continue to increase their height and the crypts become deeper. Also, nascent mesenchymal clusters carry on appearing between existing villi and stimulate production of more new villi. This process is continuously repeated in order to increase the number of villi, crypts and simultaneously the surface area of the intestine (Walton et al., 2016). As a result of such a growth pattern, reverse curves emerge continuously during crypt–villus morphogenesis and also an interesting region is observed where hinge cells appear at the crypt-villus junction with a wedge shape analogous to basal constriction (Tan and Barker, 2014; Sumigray et al., 2018) (Figure 5B). Hinge cells are required for cell-extracellular matrix adhesion in order to maintain the normal organization of crypt–villus structure, but beyond this their function remains largely unknown. Hedgehog (Hh) signaling is crucial for promoting mesenchymal cell clustering, influencing cell size, rearrangement, migration, adhesion and planar cell polarity (PCP) (Madison et al., 2005; Kolterud et al., 2009; Mao et al., 2010; Grosse et al., 2011; Walton et al., 2012). In addition, Bone morphogenetic protein (Bmp) and Wnt5a are also known to be actively involved in these processes (Rao-Bhatia et al., 2020).

### Epithelial bending of the tooth germ during odontogenesis

Odontogenesis is a dynamic and intricate process and the mammalian tooth germ progresses through a classic series of developmental stages from initial thickening to lamina, bud, cap, bell and finally crown (Peterkova et al., 2014).

**FIGURE 6:**
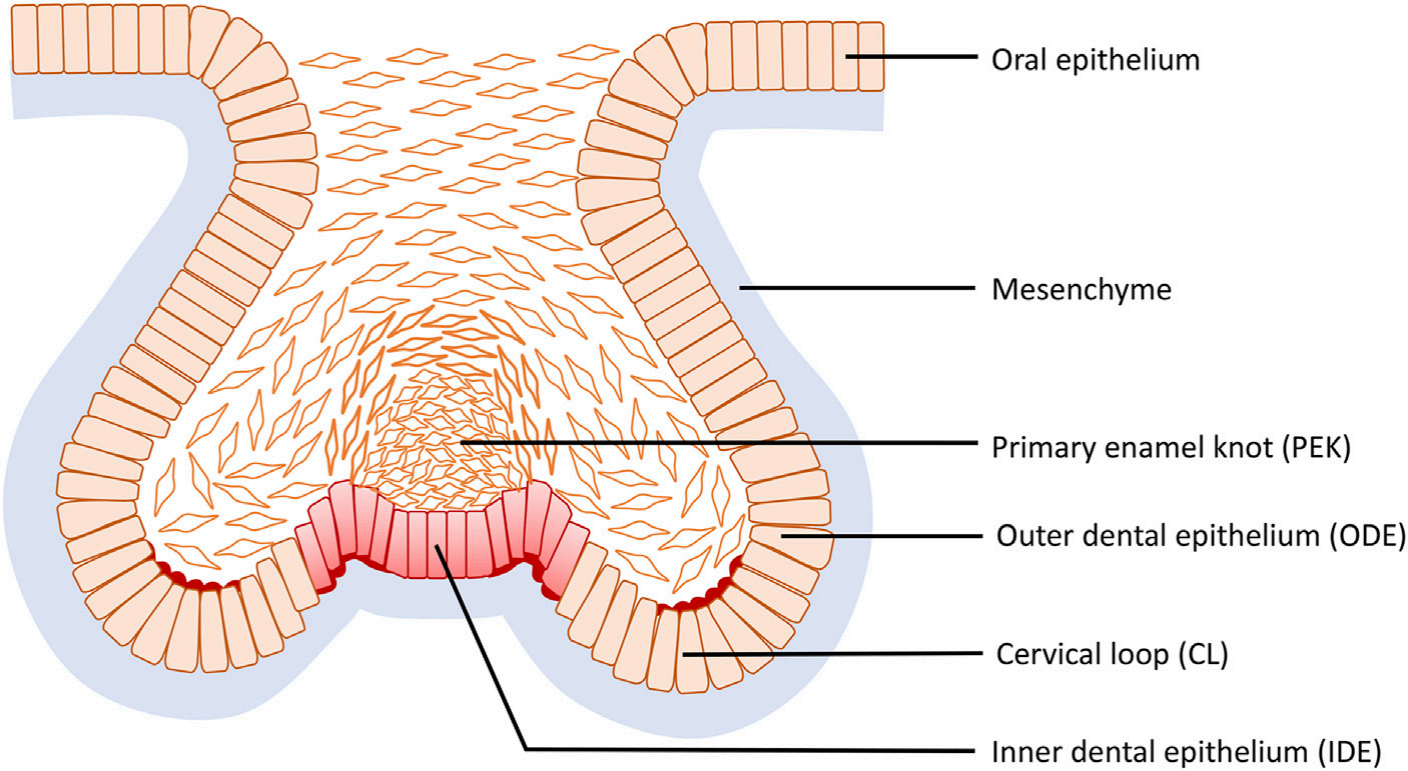
Reverse curve morphogenesis at the base of the tooth germ during tooth development. Reverse curves occur at three sites: the first is found in the neck region of the tooth bud continuing to the outline of the germ body; the second is formed by the cervical loop (CL) present bilaterally at the fold connecting the outer dental epithelium (ODE) and the lateral portion of the inner dental epithelium (IDE), while the third is situated in the primary enamel knot (EK) centered in the IDE with an invaginated outline.

During the mouse cap stage, reverse curves appear at multiple sites of the tooth germ and are clearly evident in frontal section (Figure 6). More specifically three reverse curves occur: the first is found in the neck region of the tooth bud continuing to the outline of the germ body and its formation requires the contractile canopy of the intercalating suprabasal cells; the second is formed by the cervical loop (CL) present bilaterally at the fold connecting the outer dental epithelium (ODE) and the lateral portion of the inner dental epithelium (IDE), while the third is situated centered in the primary enamel knot (EK) with an invaginated outline. As the tooth germ progresses to the early bell stage, the invaginations become the bilaterally elongated CL and the central area of the IDE, whilst the evagination transforms into the delineation of secondary EKs. The reverse curves seen in odontogenesis are limited within an individual tooth germ and do not repeat like the crypt–villus structure of the intestine. This cellular mechanism of the evagination relies on intrinsic basal constriction from the actomyosin network (Yamada et al., 2019).

As outlined above, early tooth morphogenesis from bud to early bell stage is proliferation-independent (Yamada et al., 2019). However, during later development of the tooth germ from cap to bell stage, its role remains unclear. One theory is based on the observation that the secondary EKs have low proliferation while the invaginations, i.e., CL and inter-cuspal regions, exhibit high proliferation and that this differential rate of proliferation within the stiff surrounding mesenchymal condensation drives the bending. Computational models support this interpretation of the data, but the model has yet to be fully tested experimentally (Salazar-Ciudad and Jernvall, 2010; Morita et al., 2016). Interestingly, the cap-stage molar already has a subtle invaginated contour on the underside of the EK and peaks on either side formed by basally-constricted juxta-knot cells. The surrounding mesenchyme has been suggested to be an extrinsic factor able to exert constrictive force and manipulate CL formation based on tooth germ culture following removal of mesenchyme (Alfaqeeh et al., 2013; Morita et al., 2016). However, the actual cellular mechanisms of cusp formation have not been investigated in detail. Additionally, the formation of sulci (valleys) between the cusps has received very little attention and appears from the rather limited literature to be a separate active process (Li L. et al., 2016).

Considering intrinsic mechanisms, the actomyosin system contributes to the formation of reverse curves in murine odontogenesis after the cap stage (Yamada et al., 2019). As the tooth germ develops, cell shape in the CL becomes more wedge shaped, with basal expansion and apical constriction. During the cap stage, cell shapes of the evaginated IDE adjacent to the enamel knot exhibit basal constriction and the enrichment of localized F-actin and myosin are reported at the basal side. Moreover, slice culture experiments with inhibition of FAK, an essential constituent of cytoskeleton involved in basal constriction, indicate FAK is necessary in evaginated regions during bud to cap transition (Gutzman et al., 2018; Yamada et al., 2019). Odontogenesis is an integrated consequence of genetics and biochemical signals. Primary signaling centers situated precisely in the centre of the tooth germ base, and later on also secondary signaling centers, express various molecular morphogens, including Sonic hedgehog (Shh), bone morphogenetic proteins (Bmps), and fibroblast growth factors (Fgfs) that elicit specific cell responses during development of the sophisticated reverse curves described (Kettunen et al., 2000; Liu et al., 2008).

Overall, reverse curves in invertebrate models have some universal characteristics and are derived as a result of action of the shared cellular motifs. Although they assemble into different biological structures, it is worthwhile investigating the cellular mechanisms and relative contributions to reverse curve morphogenesis. In the first instance, actomyosin-dependent apical and basal constriction is necessary for at least some epithelial invaginations and evaginations that create reverse curves. Secondly, extrinsic factors including differential proliferation and mesenchymal constraint that generate buckling pressure are significant contributors to epithelial bending.

## Recent and novel approaches applied to investigate cellular mechanisms of epithelial bending

An increased molecular understanding, new experimental techniques and rapid advances in technology have enabled the use of novel approaches to address more complex research questions. Here we summarize some recent developments in organoid culture, mathematical modelling and imaging as examples to introduce current advances in our understanding of the cellular mechanisms underlying epithelial morphogenesis and bending, especially those depicting the development of reverse curves.

### Organoid culture

Organoids are autonomous organized or reconstructed three-dimensional (3D) structures that develop from stem cells or organ-specific progenitors that aim to replicate morphology and perform similar functions to *in vivo* organs and tissues (Zachos et al., 2016). These systems have become an important tool to simulate and study tissue morphogenesis including molecular signaling and cellular dynamics (Hartl et al., 2019; Taelman et al., 2022). Tremendous progress has been made in the organ types and culture conditions, which can remarkably resemble the *in vivo* physiology more realistically than conventional two-dimensional (2D) culture.

Human intestinal organoids were the first successful human organoid culture established *in vitro* and has become the comparator for other organoid systems. They were developed from pluripotent stem cells (PSC) or from adult somatic intestinal stem cells (ISC) with culture methods using the manipulation of growth factors. This enabled a form of crypt-villus complex to be replicated, with evaginated crypts interspersed with flat villus-type epithelium forming a hinge cell region, facilitating the investigation of reverse curves in in vitro experiments (Spence et al., 2011b; Sumigray et al., 2018; Hartl et al., 2019). In addition, the intestinal organoid culture at macroscale enables the analysis of extrinsic factors which contribute greatly to the formation of reverse curves by means of tissue engineering techniques such as microcavity arrays, microfluidic chip and microprinting (Murrow et al., 2017). Genetically modified intestinal organoids could also be beneficial for the investigation of molecular mechanisms guiding epithelial bending through modification of individual gene expression. A current limitation in intestinal organoid technology is that they are constituted of only epithelial cells, despite mesenchyme being essential in epithelial morphogenesis and the formation of reverse curves.

Oral organoid culture, including the tooth germ and the CVP, have been established more recently (Gao et al., 2021). Tooth germ-like organoids can be generated by isolation of dental epithelial and mesenchymal cells or derivation from pluripotent stem cells in a specific culture system. They can also be generated by stem cell culture on a scaffold with the manipulation of odontogenic differentiation (Cai et al., 2013; Ono et al., 2017; Kim et al., 2019; Rosowski et al., 2019). Taste bud organoids can be made by implantation of isolated circumvallate papilla tissue in Matrigel with a series of growth factors (Ren et al., 2014; Aihara et al., 2015). However, these oral organoids have yet to demonstrate the reverse curves seen *in vivo* and also other limitations exist, such as shortage of cell resources, the failure of normal structural development and requirement for unique culture methods. Subsequently further refinement and development of our understanding of the cellular mechanisms involved would be a promising area of future research.

### Computational modelling

Among intestine, tooth and tongue mathematical models, tooth development is more advanced in displaying development of reverse curves than crypt-villus and papilla models. Early computational modelling of tooth development was limited to mimicking only the inner enamel epithelium (Salazar-Ciudad and Jernvall, 2002, 2010); however, more recent achievements have also demonstrated differential adhesion and growth of the three tooth germ tissues including epithelial basal layer, suprabasal layer and mesenchyme *via* a novel modelling framework of animal development (Marin-Riera et al., 2016, 2018). This model uses ubiquitous cell proliferation for the epithelium and suprabasal layers separately and applies localized proliferation of the mesenchyme according to the distance from the primary EK as well as differential adhesion between the three tissues, more closely modelling the transition of tooth development from bud to cap stage (Marin-Riera et al., 2018). Importantly, this model, in which cells were represented as solid particles (or pairs of particles in the case of columnar epithelial cells) showed that differential proliferation and adhesion could be sufficient to account for the observed morphogenesis, setting this up as a falsifiable hypothesis. The work of Yamada et al. took on this challenge and showed that *in vivo* processes not included in the model were necessary (Yamada et al., 2019). This is a perfect example of how a computational modelling cannot be directly applied to *in vivo* scenarios but nonetheless can be useful for preliminary testing of some scientific processes. Further modelling that now captures key shape change and active migration behaviours of cells may shed further light and generate hypotheses about, for example, how adhesions and cytoskeletal structures generate the right movements and forces to achieve the final organogenesis.

### Live imaging

Tracking cell shape change, movement and division at single-cell resolution can help us to understand the process of tissue deformation, cell behaviors and regulatory molecules during the morphogenesis of epithelial bending. Live imaging relies on the technique of genetic labelling, particularly mosaic labelling with fluorescent proteins such as Green fluorescent protein (GFP) and imaging traditionally with the conventional confocal fluorescence microscope and more recently with two-photon and light sheet microscopes with various fluorescent probes and reporters that have been acknowledged as the imaging of choice across multiple species and organs since cell behaviours are observed directly rather than inferred (Keller, 2013). Being inherently three-dimensional, these methods have striking advantages in demonstrating real-time spatial changes in comparison with conventional 2D histological analysis. In the study of odontogenesis and transition from cap to bell stage, live imaging of *in vitro* culture reveals the growth of the non-EK epithelium and the high potential of the CL tips when the reverse curves form *in vivo* at the base of the bell-shaped tooth germ after E14.5 (Morita et al., 2016). Further computational exploitation and processing of the long-term 3D trajectory data, including the volumetric growth rate, the deformation anisotropy, cell motility and mitotic spindle orientation, provide us with a rich 4D (3D plus time) dataset, unveiling the dynamic cellular mechanisms of reverse curves at both micro- and macroscale (Morita et al., 2016; Kim et al., 2017). The constraints with these methods are the requirements for sufficient computational power although decreasingly so as workstations become more powerful and affordable, the balance of the competing image acquisition parameters (i.e., the trade-offs between speed, sensitivity and resolution limited by the need to protect the sample against phototoxicity), and the ability to mount and image embryos and explants that faithfully undergo wild type morphogenesis.

Future prospects will involve improving these technologies but also exploring new methods for embryo, explant and organoid culture. For instance, optogenetics is a new emerging synthetic technique that has been applied to manipulate cell contractility by utilizing genetically encoded photo-activatable CRY2/CIB1 protein module (Guglielmi et al., 2015). It serves as a precise temporospatial approach that can be used to modulate Rho pathway that controls actomyosin-mediated apical constriction resulting in epithelial folding and reconstruction of the *Drosophila* mesoderm invagination (Izquierdo et al., 2018; Krueger et al., 2018). Future advances in the field of synthetic biology could provide further illustrations of the detailed cellular mechanisms that guide the developmental morphogenesis.

## Discussion

Among the two tissue bending processes that form basis for epithelial organogenesis, our understanding of invagination precedes that of evagination, which is not a simple inversion of known invagination mechanisms. Although some similar cellular motifs drive these two processes, these and the molecular signaling that control invagination and evagination cannot simply be applied from one to another. Even though these tissue bending processes are fundamental to early epithelial organogenesis, more complex epithelial morphogenesis requires elaborate coordination of different cellular motifs to act together leading to more diverse epithelial bending that also give rise to reverse curves. These reverse curves can be seen for instance in isolated (early tooth bud) or duplicated forms (CVP), in repetitive patterns (crypt-villus structure in the intestine) or in more complex forms that can give rise to unique shapes (tooth) during epithelial morphogenesis. Need for better understanding of how these structures form provides an implication for further investigations in modelling the dual cellular mechanisms involved in the assembly of the concave and convex shapes seen in epithelial reverse curves. Although, reverse curve morphogenesis through buckling has been reported in the development of *Drosophila* wing disc, the cellular mechanisms of epithelial reverse curves evident in the vertebrate and mammalian organs is likely to be more complex (Nelson, 2016).

In this review, we have summarized how previously described cellular motifs function together in both types of epithelial bending and demonstrated a clear hierarchy from intrinsic potential to external influence. By referring to these fundamental cellular motifs, we have been able to analyze the multicellular behaviors of typical reverse curves in different organs in three vertebrate models and also demonstrated how the emerging experimental methods can provide outlook for the future in the research field of temporospatial tissue morphogenesis. We have focused on cellular mechanisms and therefore present little discussion on the molecular signaling that drives these cellular changes and organogenesis. However, we know that signaling molecules including but not limited to Shh, canonical Wnt, Fgf, and Eda, govern, mediate and trigger cellular responses, diffusible signaling proteins according to their temporospatial concentration, and play important roles in growth, patterning and morphogenesis of tooth and other ectodermal organs (Ciani and Salinas, 2005; Dale et al., 2009; Rishikaysh et al., 2014; Ahtiainen et al., 2016). Combining molecular mechanisms with our understanding of cellular principles could offer a comprehensive perspective in the formation of reverse curves. However, there are currently only a few studies available investigating the development of reverse curves that limit the amount of information on the molecular environment and their differential requirements in guiding the development of reverse curves and possible competitive nature in determining cell behaviors (Sumigray et al., 2018).

Aside from organoid culture, computational modelling and time-lapse imaging, there are other promising approaches, such as genetic fate mapping and single-cell transcriptomic technologies. Making efficient use of these technologies and combining experimental capabilities can be challenging but will be necessary to gain a systems-level understanding of morphogenesis, which remains a major goal for the developmental biologists and for regenerative technologies of the future.
